# The European Larynx Organ Preservation Study [MK-3475-C44]

**DOI:** 10.3389/fonc.2024.1433238

**Published:** 2024-08-22

**Authors:** Gunnar Wichmann, Theresa Wald, Markus Pirlich, Joanna Napp, Ina Münter, Thomas Asendorf, Ralf Tostmann, Jeannette Vogt, Kathrin Vogel, Sylvia Meuret, Matthaeus Stoehr, Veit Zebralla, Nils Henrik Nicolay, Thomas Kuhnt, Peter Hambsch, Orlando Guntinas-Lichius, Jens Peter Klußmann, Susanne Wiegand, Andreas Dietz

**Affiliations:** ^1^ Clinic for Otorhinolaryngology and Head and Neck Surgery, Department of Head Medicine and Oral Health, University of Leipzig, Leipzig, Germany; ^2^ The Comprehensive Cancer Center Central Germany, Leipzig University Hospital, Leipzig, Germany; ^3^ Clinical Trial Unit, University Medical Center Goettingen, Göttingen, Germany; ^4^ Department of Medical Statistics, University Medical Center Goettingen, Goettingen, Germany; ^5^ Clinic for Radiation Oncology, University Hospital Leipzig, Leipzig, Germany; ^6^ ENT Department, Jena University Hospital, Jena, Germany; ^7^ The Comprehensive Cancer Center Central Germany, Jena University Hospital, Jena, Germany; ^8^ Department of Oto-Rhino-Laryngology Head and Neck Surgery, University of Cologne, Cologne, Germany; ^9^ Department of Otorhinolaryngology, Head and Neck Surgery, Christian-Albrechts-University Kiel, Kiel, Germany

**Keywords:** head and neck squamous cell carcinoma (HNSCC), larynx and hypopharynx cancer (LHSCC), larynx organ preservation (LOP), total laryngectomy (TL), inductionchemotherapy (IC), randomized controlled trial (RCT), larynx organ function, immune checkpoint blockade PD-1:PD-L1 axis

## Abstract

The European Larynx Organ Preservation Study (ELOS; NCT06137378) is a prospective, randomized, open-label, two-armed parallel group controlled, phase II multicenter larynx organ preservation (LOP) trial in locoregionally advanced (LA) stage III, IVA/B head and neck squamous cell carcinoma of the larynx or hypopharynx (LHSCC) amenable for total laryngectomy (TL) with PD-L1 expression within tumor tissue biopsy, calculated as CPS ≥ 1. Induction chemotherapy (IC) with docetaxel and cisplatin (TP) followed by radiation will be compared to TP plus PD-1 inhibition by pembrolizumab (MK-3475; 200 mg i.v. starting day 1 q3w for 17 cycles). After a short induction early response evaluation (ERE) 21 ± 3 days after the first cycle of IC (IC-1), responders achieving endoscopic estimated tumor surface shrinkage (ETSS) ≥30% will get an additional two cycles of IC followed by intensity-modulated radiotherapy 70–72 Gy (EQD2/α/β = 10) aiming at LOP. Nonresponders (ETSS < 30% or progressing disease) will receive TL and bilateral neck dissection followed by postoperative radiation or chemoradiation as recommended by the clinic’s multidisciplinary tumor board. Pembrolizumab treatment will be continued in the intervention arm regardless of ETSS status after IC-1 in both responders and laryngectomized nonresponders, independent of subsequent decisions on adjuvant therapy after TL.

**Clinical Trial Registration:**

clinicaltrials.gov, identifier NCT06137378.

## Introduction

The European Larynx Organ Preservation Study (ELOS) (1) is a prospective, randomized, open-label, two-armed parallel group controlled, phase II multicenter larynx organ preservation (LOP) trial in locoregionally advanced (LA) stage III, IVA/B squamous cell carcinoma of the larynx or hypopharynx (LHSCC) with PD-L1 expression within tumor tissue biopsy, calculated as CPS ≥ 1, amenable for total laryngectomy (TL). Large stage II hypopharyngeal LHSCCs only resectable by total laryngopharyngectomy are also eligible. Induction chemotherapy (IC) with docetaxel and cisplatin (TP) followed by radiation will be compared to TP and additional PD-1 inhibition. Patients will be selected after a short induction early response evaluation (ERE) after the first cycle of IC (IC-1) aiming at LOP by additional two cycles of IC followed by radiotherapy (RT) for responders achieving endoscopic estimated tumor surface shrinkage (ETSS) ≥ 30% (1–3). Nonresponders (ETSS < 30% or progressing disease) will receive TL and neck dissection (ND), preferably ipsilateral and contralateral selective neck dissection (SND), followed by postoperative radiation (PORT) or cisplatin-based concurrent chemoradiation (PORCT) according to the recommendation of the clinic’s multidisciplinary tumor board (MDTB). However, patients randomized into the intervention arm will receive pembrolizumab (MK-3475) 200 mg i.v. starting at day 1 and in a 3-week cycle (q3w) for up to 17 cycles (12 months). Treatment with pembrolizumab will continue in the experimental arm regardless of ETSS status after IC-1 in both responders and laryngectomized nonresponders, independent of subsequent decisions on adjuvant therapy after TL.

The primary objective of ELOS is to compare laryngectomy-free survival (LFS) achieved by adding pembrolizumab (KEYTRUDA^®^) to standard treatment and LFS after standard treatment according to the DeLOS-II protocol in advanced LHNSCC curable by TL. Hypothesis: Adding PD-1 inhibition by pembrolizumab to organ preservation chemoradiation treatment improves LFS compared to standard treatment according to the DeLOS-II protocol.

The secondary objectives are to compare quality of swallowing (QoS) assessed by fiber-endoscopic assessment of swallowing (FEES), event-free survival (EFS), and overall survival (OS) achieved by adding pembrolizumab to standard treatment and QoS, EFS, and OS after standard treatment according to the DeLOS-II protocol in advanced LHSCC.

In general, the main interest in trials focusing on improving quality and degree of LOP is late functional (in particular "swallowing") outcome. Current instruments assessing health-related QoL are less meaningful than direct objective assessment of swallowing utilizing physical examination like FEES. FEES is a well-approved and reliable method and allows clear scoring of QoS for instance by applying the Rosenbek Scale (4). Therefore, the investigators decided to avoid any questionnaires for this assessment including those approved for use in head and neck cancer, as they fail in specifically addressing the main study outcome, functional LOP.

Hypothesis: Adding PD-1 inhibition by pembrolizumab to organ preservation chemoradiation treatment improves QoS, EFS, and OS compared to standard treatment according to the DeLOS-II protocol. EFS events are defined as any event interfering with either proper larynx organ function (independent of the cause, tumor or treatment related), relapse (local, loco-regional, or distant), or death. However, TL in nonresponders with ETSS < 30% is per-protocol defined treatment, and early salvage TL is not considered as an event in the EFS analyses with alternative EFS definition.

Preceding ELOS, the German multicenter randomized phase-II LOP trial DeLOS-II (2) was performed to investigate the impact of cetuximab added to induction chemotherapy and radiation on LFS in LA LHSCC. Untreated patients with stage III/IV LHSCC amenable to TL were randomized to three cycles of IC with TP (docetaxel and cisplatin 75 mg/m²/day for day 1) followed by RT without (A) or with (B) standard dose cetuximab for 16 weeks. (The initially used TPF regimen 5-FU 750 mg/m²/day for days 1–5 was replaced with TP; this reduced acute toxicity and had no impact on response rate and outcome (2).) Response to the first IC cycle with ETSS ≥ 30% was used to define early responders; early salvage TL was recommended to nonresponders. The primary objective (24-month LFS above 35%) was equally met by arms A (40/85, 47.1%) and B (41/88, 46.6%). The 24-month OS rates were 68.2% and 69.3% (2).

The PD-1:PD-L1 pathway is an attractive target for therapeutic intervention for LOP in LA LHSCC that can only be cured by surgery with TL and ND followed by RT or radiochemotherapy. However, LA LHSCC requires an immediate reduction of bulk tumor masses that can be best achieved by an IC with cisplatin (P) combined with 5-fluorouracil (F) or a taxane (T), most preferably docetaxel. The triple combination TPF, however, puts LHSCC patients at increased risk for serious adverse events and fatal outcome in up to 8% of patients (2). Therefore, and as shown in DeLOS-II, the omission of F and the use of TP for IC reduces side effects and the number of fatal events, by maintaining the efficacy of IC (2). Moreover, partial response according to ETSS ≥ 30% achieved through TP had the highest positive predictive value for oncologic safety and successful LOP in this patient population.

## Methods

### Study design

**Figure 1:**
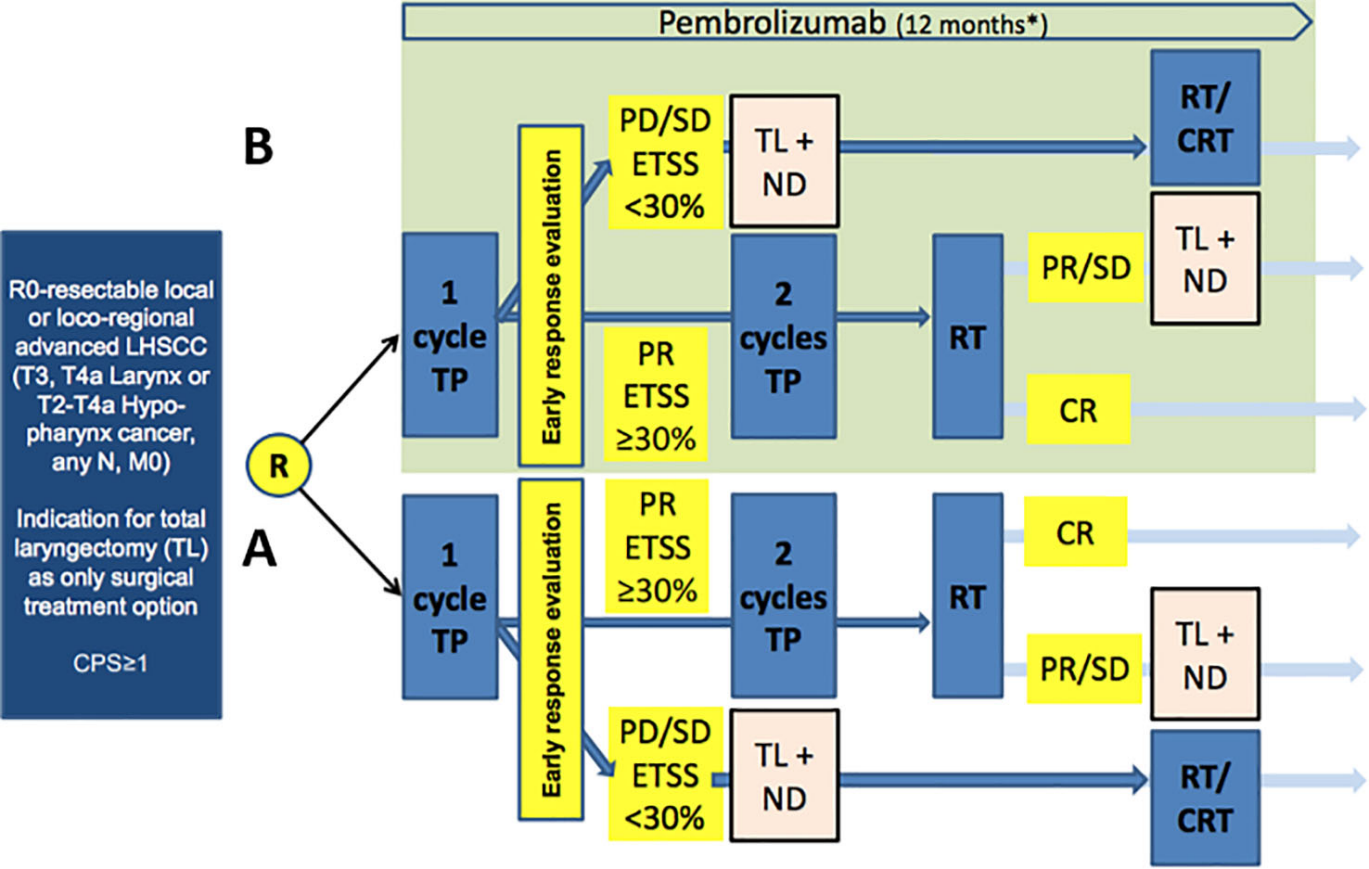
Study design of European Larynx Organ Preservation Study (ELOS) [MK-3475-C44] (NCT06137378), a randomized controlled trial comparing the outcome of patients in arm A (control) treatment according to the medication and radiation protocol as in the DeLOS-II LOP trial (2) with the same treatment plus additional pembrolizumab (over 12 months) in the experimental arm (B; light green). CPS: combined positive score; R, randomization; TP, induction chemotherapy utilizing T, docetaxel, P, cisplatin (75 mg/m^2^ each per cycle); Early response evaluation (ERE) according to DeLOS-II criteria: PR, partial response ≥ 30% endoscopic tumor surface shrinkage (ETSS) after one cycle; PD/SD, progressing disease or insufficient response < 30% ETSS; TL, total laryngectomy; ND, neck dissection; RT, radiotherapy; CRT, concomitant cisplatin-based chemo-radiotherapy.

ELOS is a prospective, randomized, open-label, two-armed parallel group controlled, phase II multicenter LOP trial with randomization in a 1:1 ratio into standard arm versus investigational arm receiving pembrolizumab with a flexible follow-up of 24–48 months. The design of ELOS is shown in **Figure 1**.

Arm A is the standard-treatment arm with no intervention (control). Patients randomized into arm A will receive a short induction treatment with a single cycle TP (IC-1) (T = docetaxel 75 mg/m^2^ i.v. day 1, P = cisplatin 75 mg/m^2^ i.v. day 1). Response evaluation will be performed in week 4 (day 21 ± 3 days) after IC-1 by endoscopic estimation of tumor-surface shrinkage (ETSS) to select nonresponders for early TL. Consequently, nonresponders will undergo TL and receive adjuvant RT or chemo-radiotherapy (CRT) according to the decision of the clinic’s MDTB. Responders with ETSS ≥ 30% receive additional two cycles of TP (IC-2 in week 4 ± 3 days and IC-3 in week 7 ± 3 days; same doses as IC-1) followed by RT starting at week 11. Radiotherapy (RT) will be carried out using intensity-modulated radiotherapy (IMRT) with a total dose of 70–72 Gy (EQD2/α/β = 10) to all macroscopic tumor localizations. The elective neck nodal levels should be treated with 45–54 Gy depending on the risk of recurrence.

Arm B is the experimental arm receiving the only intervention, KEYTRUDA^®^ (pembrolizumab). The treatment is the same as that for patients randomized into the standard arm A plus additional i.v. application of pembrolizumab in a 3-week cycle (q3w) of 200 mg each, starting on day 1 for up to 17 cycles (12 months). The treatment with pembrolizumab will continue in the experimental arm regardless of ETSS status after IC-1 in both responders and laryngectomized nonresponders, independent of subsequent decisions on adjuvant therapy with RT or CRT after TL.

### Study population

Based on statistical considerations according to LFS in DeLOS-II, a sample of 140 patients with LA LHSCC resectable only by TL was planned to be recruited according to the following selection criteria.

### Inclusion criteria

Participants are eligible to be included in the study only if all of the following criteria apply:

1. Male and female participants who are at least 18 years of age on the day of signing informed consent with histologically confirmed diagnosis of squamous cell carcinoma (SCC) of the larynx or hypopharynx according to the decision of the MDTB suitable for TL can be enrolled in this study.
2. Stage II (large cT2 cN0 hypopharynx cancer only) and III, IVA, or IVB, whenever clear resection margins R0 > 5 mm can be achieved and no radiologic signs of extranodal extension of neck nodes are present.
3. Have provided newly obtained excisional biopsy of a tumor lesion not previously irradiated. Formalin-fixed, paraffin-embedded (FFPE) tissue blocks are preferred to slides.
4. PD-L1-expression* within the tumor biopsy, CPS ≥ 1.
5. Male participants
  A male participant must agree to use a contraception as detailed in Appendix 3 of this protocol during the treatment period and for at least 120 days after the last dose of study treatment and refrain from donating sperm during this period.
6. Female participants
  A female participant is eligible to participate if she is not pregnant (see Appendix 3), not breastfeeding, and at least one of the following conditions applies:
  1. Not a woman of childbearing potential (WOCBP) OR.
  2. A WOCBP who agrees to follow the contraceptive guidance during the treatment period and for at least 120 days after the last dose of study treatment.
7. Have an Eastern Cooperative Oncology Group (ECOG) performance status of 0 to 1. Evaluation of ECOG is to be performed within 7 days prior to the date of allocation/randomization.
8. Have adequate organ function as defined in Table 4 of the protocol (**Table 1**). Specimens must be collected within 10 days prior to the start of study treatment.

**Table 1:** Adequate organ function laboratory values for ELOS-eligible LHSCC patients.

System	Laboratory value
Hematological	
Absolute neutrophil count (ANC)	≥1,500/µL
Platelets	≥100,000/µL
Hemoglobin	≥9.0 g/dL or ≥5.6 mmol/L^a^
Renal	
Creatinine OR Measured or calculated ^b^ creatinine clearance (GFR can also be used in place of creatinine or CrCl)	≤1.5 × ULN OR ≥30 mL/min for participant with creatinine levels >1.5 × institutional ULN
Hepatic	
Serum total bilirubin	≤1.5 × ULN OR direct bilirubin ≤ULN for participants with total bilirubin levels >1.5 × ULN
AST (SGOT) and ALT (SGPT)	≤2.5 × ULN
Coagulation	
International normalized ratio (INR) OR prothrombin time (PT) Activated partial thromboplastin time (aPTT)	≤1.5 × ULN unless participant is receiving anticoagulant therapy as long as PT or aPTT is within therapeutic range of intended use of anticoagulants

ALT (SGPT), alanine aminotransferase (serum glutamic pyruvic transaminase); AST (SGOT), aspartate aminotransferase (serum glutamic oxaloacetic transaminase); GFR, glomerular filtration rate; ULN, upper limit of normal. ^a^ Criteria must be met without erythropoietin dependency and without packed red blood cell (pRBC) transfusion within last 2 weeks. ^b^ Creatinine clearance (CrCl) should be calculated per institutional standard.

* As predetermined by the EMA, the assessment of PD-L1 status has to be performed according to the guidelines for first-line treatment of head and neck cancer with pembrolizumab by using the CE-certified PD-L1 IHC 22C3 pharmDx assay (Agilent).

### Exclusion criteria

Participants are excluded from the study if any of the following criteria apply:

1. A WOCBP who has a positive urine pregnancy test within 72 h prior to receiving the first dose of study medication (see Appendix 3). If the urine test is positive or cannot be confirmed as negative, a serum pregnancy test will be required.
2. Has received prior therapy with an anti-PD-1, anti-PD-L1, or anti PD-L2 agent or with an agent directed to another stimulatory or co-inhibitory receptor on T or NK cells (e.g., CTLA-4, OX 40, and CD137).
3. Has received prior systemic anti-cancer therapy including investigational agents.
4. Has received prior RT.
5. Has received a live vaccine or live-attenuated vaccine within 30 days prior to the first dose of study drug. Administration of killed vaccines is allowed.
6. Is currently participating in or has participated in a study of an investigational agent or has used an investigational device within 4 weeks prior to the first dose of study intervention.
7. Has a history of a second malignancy, unless potentially curative treatment has been completed with no evidence of malignancy for 2 years.
8. Has known distant metastases including active CNS metastases and/or carcinomatous meningitis.
9. Has severe hypersensitivity (≥ grade 3) to pembrolizumab and/or any of its excipients.
10. Has active autoimmune disease that has required systemic treatment in the past 2 years (i.e., with the use of disease-modifying agents, corticosteroids, or immunosuppressive drugs). Replacement therapy (e.g., thyroxine, insulin, or physiologic corticosteroid replacement therapy for adrenal or pituitary insufficiency, among others) is not considered a form of systemic treatment and is allowed.
11. Has a history of (non-infectious) pneumonitis/interstitial lung disease that required steroids or has current pneumonitis/interstitial lung disease.
12. Has an active infection requiring systemic therapy.
13. Has a known history of human immunodeficiency virus (HIV) infection. No HIV testing is required unless mandated by local health authority.
14. Has a known history of hepatitis B [defined as hepatitis B surface antigen (HBsAg) reactive] or known active hepatitis C virus [defined as HCV RNA (qualitative) is detected] infection. Note: no testing for hepatitis B and hepatitis C is required unless mandated by the local health authority.
15. Has a known history of active TB (*Bacillus tuberculosis*).
16. Has a history or current evidence of any condition, therapy, or laboratory abnormality that might confound the results of the study or interfere with the subject's participation for the full duration of the study, or is not in the best interest of the subject to participate, in the opinion of the treating investigator.
17. Has known psychiatric or substance abuse disorders that would interfere with cooperation with the requirements of the trial.
18. Is pregnant or breastfeeding or expecting to conceive or father children within the projected duration of the study, starting with the screening visit through 120 days after the last dose of trial treatment.
19. Has had an allogenic tissue/solid organ transplant.
20. Has a known intolerance to one of the substances administered during treatment including, e.g., antiemetics, or any other component of concurrent auxiliary medication (e.g., docetaxel or cisplatin).

### Statistical methods

#### Estimation of sample size

Focusing on analyses of the impact of pembrolizumab, we propose a design as shown in the study schema with a 1:1 randomization ratio (at least 70 patients each arm). The patients in the control arm will be treated with the same doses of IC and IMRT as in DeLOS-II standard arm A (2) allowing for reliable comparison (outside protocol). In DeLOS-II, the LFS at 24 months was 47%. A delta of 19% in LFS at 24 months attributable to additional pembrolizumab resulting in 66% LFS would be of clinical significance. As ELOS is an early clinical trial, the one-sided significance level is chosen higher than the usual 2.5% level for confirmatory phase III trials. Based on calculations performed in nQuery 8, a sample size of 70 per group, with a total number of events of 72 required, with α = 0.05 (one-sided significance level) in an exponential maximum likelihood test of equality of survival curves (according to the log-rank test) will have 81.1% power to detect a difference between the exponential parameters 0.378 (arm A, control group) and 0.208 (arm B, pembrolizumab). This corresponds to a constant hazard ratio (HR) = 1.817 and considers a common exponential dropout rate of 0.05 and flexible follow-up per patient, as described.

#### Randomization

Patients are randomized in a 1:1 ratio (70 treatment, 70 control) by minimization considering stratification by study center, localization of the primary lesion (larynx versus hypopharynx), and involvement of neck nodes (N0/N1 versus N2/N3 disease). A web-based solution for randomization (SecuTrial^®^) has been implemented.

#### Statistical principles

ELOS is an early clinical trial (phase II) to assess possible effect sizes. One-sided *p*-values lower than 5% will be considered significant. Both 90% and 95% confidence intervals (CIs) are reported for endpoints. All statistical analyses will test for superiority of the treatment arm adding pembrolizumab at a one-sided significance level of 5%. No statistical interim analyses are planned. The final analysis will be conducted after database lock that is scheduled to be exactly 24 months after randomization of the last patient.

The primary efficacy analysis as well as secondary efficacy analyses are based on the full analysis set (FAS), defined as all patients who were randomized. The FAS is analyzed following the intention-to-treat principle, in which every patient is analyzed as randomized.

Safety analyses are based on the safety analysis set (SAS) including all randomized patients who received at least one dose of study medication or standard of care.

Sensitivity analyses are performed on all patients completing the study adherent to the study protocol, referred to as the per-protocol (PP) population. The PP population consists of all patients without major protocol deviations.

#### Statistical analyses

Time-to-event endpoints (LFS, EFS, and OS) are analyzed using a Cox proportional hazards regression model with treatment and the stratification variables of the randomization [including study site, sex (male versus female), localization of the primary lesion (larynx versus hypopharynx), and involvement of neck nodes (N0/N1 versus N2/N3 disease)] as well as comorbidity according to the Charlson comorbidity index (5) as covariates. Analyses are conducted using the package survival from R with the corresponding function *coxph*. If the number of events is too small to fit the described model, a reduced model (i.e., including only the most important prognostic covariates assessed in univariate analyses) will be used. The treatment effect will be reported as HR with 90% and 95% CI and a *p*-value for the two-sided null hypothesis H0: HR = 1. The primary endpoint and the secondary endpoints (**Table 2**) will be visualized as Kaplan–Meier curves. Sensitivity analyses will explore the robustness of these analyses. Missing data from time-to-event endpoints are dealt with using right censoring.

**Table 2:** Objectives and related endpoints of the ELOS trial.

	Objective	Endpoint
Primary	Comparison of laryngectomy-free survival (LFS) between treatment groups	Hazard ratio in LFS between treatment groups
Secondary	Comparison of overall survival (OS) between treatment groups	Hazard ratio in OS between treatment groups
	Comparison of quality of swallowing (QoS) by fiber-optic endoscopic evaluation of swallowing (FEES)	Difference in proportions of patients in FEES categories assessed by Rosenbek scale at baseline and at months 6 and 24
	Comparison of event-free survival (EFS) between treatment groups	Hazard ratio in EFS between treatment groups
	Comparison of event-free survival (EFS) between treatment groups – alternative definition of EFS†	Hazard ratio in EFS between treatment groups
Safety	Compare safety of medication between treatment groups	Summary statistics of AE, SAE, and laboratory assessments

† As total laryngectomy (TL) in nonresponders with ETSS < 30% is per-protocol defined treatment, early salvage TL is not considered as an event in these analyses.

The Rosenbek scale is compared between treatment groups at 6 and 24 months using ordinal regression (package ordinal) with baseline, treatment, visit, and treatment-by-visit interaction. Proportions of patients at different score levels are reported with 95% CI. Shift differences from baseline and between treatments at 6 and 24 months are estimated using marginal means (package *emmeans*). Development of the Rosenbek scale over time is visualized using alluvial plots. A sensitivity analysis using mixed-models repeated measures (MMRM) is also conducted for the Rosenbek scale, with random patient intercept to account for dependent observations. Estimated marginal means are reported for group differences at 6 and 24 months. Missing data from the Rosenbek scale are dealt with using multiple imputation methods.

Safety analyses will follow standard procedures for the reporting of adverse events. We report safety analysis results as frequencies (percentages) by treatment group for the SAF. Descriptive reporting of laboratory parameters follows ICH E3 guidelines.

## Discussion

ELOS investigates as a randomized controlled trial the potential benefit of PD-1 targeting by pembrolizumab added during the complete course of three cycles of TP-IC and RT and up to 12 months in total by comparing LFS and OS with the control arm utilizing three cycles of TP-IC (as in DeLOS-II). Each 3-week TP-IC cycle will start with docetaxel and cisplatin each with 75 mg/m² at day 1. Immediately after the third cycle of TP, RT will follow and a total dose of 70–72 Gy (EQD2/α/β = 10) will be administered by IMRT, as this protocol was shown to be safe and achieves good outcome (2). Nonresponders defined by ETSS < 30% after IC-1 will be recommended to receive early TL followed by adjuvant radio(chemo)therapy.

Additional pembrolizumab in chemoradiation protocols does not synergistically augment or stimulate hematologic or tissue affecting toxicity. The profile is more related to autoimmune effects like pneumonitis, colitis, and hypophysitis. Data from KEYNOTE-012 (7) suggest a moderate toxicity profile as described below.

The trade-off between expected increase of efficacy and moderate toxicity does not limit the meaning and rationale of the trial from a clinical and ethical view.

According to KEYNOTE-048, response to pembrolizumab monotherapy or added to platinum-based chemotherapy in first-line chemotherapy for recurrent and/or metastatic (R/M) HNSCC without curative treatment option was superior in patients with tumors expressing PD-L1 (CPS ≥ 1) and increased their survival (6). Consequently, the European Medicines Agency’s (EMA’s) Committee for Medicinal Products for Human Use (CHMP) adopted a positive opinion recommending on 17 October 2019 a change to the terms of the marketing authorization for pembrolizumab as monotherapy or in combination with platinum and 5-fluorouracil (F) chemotherapy. Since then, pembrolizumab is indicated for the first-line treatment of R/M HNSCC in adults whose tumors express PD-L1 with a CPS ≥ 1. Therefore, a prerequisite for using pembrolizumab in HNSCC in the EU is having a CPS ≥ 1 as stated in the inclusion criteria mentioned above.

Since PD-1 inhibition with pembrolizumab proved to be highly effective in second-line R/M HNSCC treatment (7–10) and first-line R/M HNSCC treatment (6), this new immune oncological checkpoint inhibitor therapy should be investigated in the curative setting including LOP trials. So far, pembrolizumab is in trials for its possible integration into primary standard therapy concepts for curative treatment of local advanced HNSCC (11, 12). At this time, final data for KEYNOTE-412 (11) and for ADRISK (12) and any data in this new indication (LOP) are not available yet. However, the results reported for KEYNOTE-412 (NCT03040999) at clinicaltrials.gov, despite failing to demonstrate superior event-free survival through added pembrolizumab, showed encouraging data (11). KEYNOTE-412 missed the pre-specified boundary of 0.0242 required for statistical significance according to the *p*-value of 0.0429 in the per-protocol defined stratified analysis. However, the HR of 0.83 with a two-sided 95% CI of 0.68 to 1.03 in a more heterogeneous population of LA HNSCC in a different setting (administered during definitive cisplatin-based radiochemotherapy) does not provide sufficient arguments against the use of pembrolizumab in an LOP trial. The LOP trial ELOS will be conducted according to the DeLOS-II protocol and hence is a very different therapy regimen as it utilizes a prolonged induction phase (up to three cycles of pembrolizumab simultaneous to TP-IC before the first irradiation). Indeed, ELOS will recommend early TL to all patients without sufficient ETSS < 30%, whereas only responders will receive three cycles of IC with cisplatin and additional docetaxel. Moreover, compared to KEYNOTE-412, ELOS will accrue a rather homogeneous cohort of HNSCC patients with narrower HNSCC characteristics. Indeed, neoadjuvant and adjuvant pembrolizumab achieved stronger responses in a higher frequency as observed in the first-line setting (14), and the combination of PD-1 immune-checkpoint blockade (ICB) with either neoadjuvant chemotherapy (15) or CTLA-4 ICB with ipilimumab in the IMCISION trial (16) demonstrated a high response rate including pathological responses without impairing resectability (16). Added docetaxel should further increase immunogenicity and anti-tumoral immune responses unleashed by pembrolizumab, and pembrolizumab indeed demonstrated high efficacy and safety in combination with paclitaxel and cisplatin in a single-arm phase II trial (17). Retrospective analyses of rather small cohorts treated with PD-1 ICB confirm these results in LA LHSCC (18) and LA hypopharyngeal cancer (13). Because of the encouraging data on survival mentioned above and the favorable PD-L1 expression as an inclusion criterion, pembrolizumab will also be continued in the experimental arm regardless of the response to IC with ETSS depending mostly on TP. All these reports on pembrolizumab in the neoadjuvant and adjuvant setting concluded that randomized controlled trials are urgently required to validate these rather general findings. Moreover, as all these reports so far available did not include the standardized evaluation of larynx function and QoS in particular, we will conduct the randomized controlled ELOS trial to show increased LFS and QoS in LA LHSCC.

### Benefit–risk assessment

#### Expected benefit

In approximately one-third of patients, standard TP IC followed by RT is unsuccessful, and their larynx must be removed via TL. Based on the current literature (2, 3, 5–10, 12–18), the investigators expect that pembrolizumab administration will facilitate LOP and OS by allowing more patients to preserve their larynx and a permanent prevention of cancer recurrence even in laryngectomized nonresponders by administering pembrolizumab over a period of 1 year. In addition to the benefit for the patients included, the results of the study may help to contribute to the future treatment of patients with LA LHSCC.

#### Risks and burdens

The investigational medicinal product pembrolizumab may have side effects that may or may not increase the risk for adverse events of standard IC + RT therapy. However, there are no reports about an increased risk for adverse events exceeding those expected from TP alone (13–15, 17, 18). Since pembrolizumab has a marketing authorization in Germany as well as worldwide for the treatment of various tumor diseases including HNSCC, many side effects are already known (6–9, 11, 13, 17, 18). The most common side effects of pembrolizumab are diarrhea, nausea, itching, rash, fatigue, and inflammation in the body caused by the highly activated immune system (autoimmune reactions), all mostly at grade 1 or 2 according to CTCAE (19). In addition, the as-yet untested combination of pembrolizumab with docetaxel and cisplatin may cause additional adverse effects that have not been previously reported. However, we expect these to be in the range reported for pembrolizumab combined with paclitaxel and cisplatin (16–18) and not exceeding those observed in the DeLOS-II trial (2). However, treatment is discontinued if there is progression of the disease, if the patient cannot tolerate the treatment, or if the patient so desires.

All examinations performed as part of the study are examinations that are usually performed as part of the standard treatment of the disease and do not pose any identifiable additional risk. The potential benefit of participating in ELOS should be considered higher than the potential risk.
